# Towards Autonomous Driving: Technologies and Data for Vehicles-to-Everything Communication

**DOI:** 10.3390/s24113411

**Published:** 2024-05-25

**Authors:** Vygantas Ušinskis, Mantas Makulavičius, Sigitas Petkevičius, Andrius Dzedzickis, Vytautas Bučinskas

**Affiliations:** Department of Mechatronics, Robotics and Digital Manufacturing, Vilnius Gediminas Technical University, LT-10105 Vilnius, Lithuania; vygantas.usinskis@vilniustech.lt (V.U.); m.makulavicius@vilniustech.lt (M.M.); sigitas.petkevicius@vilniustech.lt (S.P.); andrius.dzedzickis@vilniustech.lt (A.D.)

**Keywords:** sensors, vehicle technologies, machine learning, communications

## Abstract

Autonomous systems are becoming increasingly relevant in our everyday life. The transportation field is no exception and the smart cities concept raises new tasks and challenges for the development of autonomous systems development which has been progressively researched in literature. One of the main challenges is communication between different traffic objects. For instance, a mobile robot system can work as a standalone autonomous system reacting to a static environment and avoiding obstacles to reach a target. Nevertheless, more intensive communication and decision making is needed when additional dynamic objects and other autonomous systems are present in the same working environment. Traffic is a complicated environment consisting of vehicles, pedestrians, and various infrastructure elements. To apply autonomous systems in this kind of environment it is important to integrate object localization and to guarantee functional and trustworthy communication between each element. To achieve this, various sensors, communication standards, and equipment are integrated via the application of sensor fusion and AI machine learning methods. In this work review of vehicular communication systems is presented. The main focus is the researched sensors, communication standards, devices, machine learning methods, and vehicular-related data to find existing gaps for future vehicular communication system development. In the end, discussion and conclusions are presented.

## 1. Introduction

**Figure 1:**
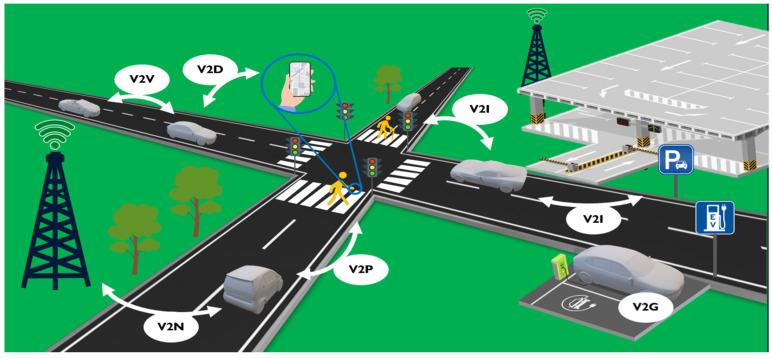
Vehicular communication elements: V2V—vehicle-to-vehicle; V2I—vehicle-to-infrastructure, V2P—vehicle-to-pedestrian, V2D—vehicle-to-device, V2N—vehicle-to-network, and V2G—vehicle-to-grid communications.

Intelligent transport systems (ITSs) define progressive topics of connected cars, connected automated driving, and vehicular communication systems which are expected to be game changers for future traffic mobility with further technological developments [1,2]. The main concern of ITSs is the increase in road safety and security by minimizing or fully avoiding human errors through the development of autonomous vehicles (AVs) [3]. It is unlikely that Avs can achieve their full potential without automating the vehicle’s communication with surrounding objects. This can be achieved through technological improvements in sensors that can sense the surrounding environment based on physical stimuli and a range of communication equipment that transmits collected and/or pre-processed data to nearby road users to ensure an efficient traffic cycle. Communication quality is one of the critical factors that determine the development of ITSs. According to [4], recent studies have analyzed and developed road safety and security in terms of latency and reliability. Research has also concluded that ITSs, because of wireless data transmission, encounter various attacks, e.g., signal hacking, that could lead to reduced autonomous driving performance. Therefore, the main communication attributes such as data authentication, availability, confidentiality, and real-time constraints must be taken into account. The concept of vehicular communication systems in the common literature is known more as vehicle-to-everything (V2X) communication, which has a wide range and covers different traffic elements, as shown in Figure 1.

These different elements of V2X communication are essential for autonomous driving to make it safe and robust. The complexity of autonomous driving is defined in terms of automation levels, which can be specified for a particular V2X element and are represented in Table 1, as described in previous research [5,6].

Each element of V2X has specific advantages, problems, and limitations. One of the main communications elements of V2X that has attracted attention is connectivity between vehicles (V2V). The main task and challenge of this connectivity is to enable unlimited data exchange in real-time without additional means [7]. It is believed that the achievement of this aim will enhance and replace traditional forms of data exchange in traffic with different wireless communications. For example, many turn to AI methods for developing improved wireless communications systems with enhanced optimization and security changing human-based linear rules to AI-based non-linear rules.

Research presented in [8] has pointed out four main V2V-related applications: traffic management, road safety, direction and route optimization, and driver assistance. Traffic management can be implemented using shared communication systems between vehicles to avoid high traffic and congestion and to optimize the schedule of traffic lights to reduce average delays. For road safety applications, the main concern is to prevent and reduce the number of road accidents, which are represented in terms of communication delays. Direction and route can be optimized by analyzing road and weather conditions. Driver assistance, also known as Advanced Driver Assistance Systems (ADASs), can be used to improve, automate, or adapt some or all of the tasks depending on vehicle operation, e.g., braking or avoiding collisions. One of the examples of V2V communication is platooning, where connected and autonomous vehicles can coordinate their driving speed to reduce vehicles’ air resistances by optimizing the distance between them [9].

Vehicles enroute face not only other vehicles but also surrounding infrastructure such as traffic lights, road signs, communication antennas, buildings, bridges, etc. Communication with such objects is referred to as vehicle-to-infrastructure (V2I) connectivity. V2I connectivity can be divided into two big research fields according to the raised challenges and required equipment. These sub-fields are: surrounding or road infrastructure [10] and smart parking systems [11]. The main concern of surrounding infrastructure (outside) is that can be influenced by the environment and weather [12], whereas smart parking systems (mostly inside) are the signals throughput across dense constructions [13]. They can be improved using additional equipment. In the road infrastructure, sensors like cameras, radars, and other infrastructure like road signs or weather stations are used to broadcast information, e.g., about speed limits and weather conditions [14]. In smart parking systems, for example, proximity sensors [15] and Radio-Frequency Identification (RFID) [16] tags are used to identify and broadcast the data about a vehicle or parking lot status.

Even more vehicles, like battery electric vehicles (BEV) and plug-in hybrid vehicles (PHEV), are becoming prevalent in traffic, and additional infrastructure of charging stations is necessary in parking places and at homes. Therefore, the information regarding available charging stations or needed load is important. This particular case is called vehicle-to-grid (V2G) communication, in which the main concern is to balance charging loads, e.g., by transferring the energy from the most charged cell to the least charged, in parking systems [17] or even at homes (vehicle-to-home (V2H)) [18] based on data exchange with electric vehicles (EV), thus reducing bill costs.

Another aspect of V2X communication is vehicle-to-pedestrian (V2P) connectivity, which is also an important part of the traffic, and the main concern is to ensure the safety of both parties [19]. This communication uses on-board sensors in the vehicle, like LiDAR (light detection and ranging), radars, or cameras to warn the drivers of some detected obstacles, e.g., in their way and blind spots, or automatically bypassing them, thus reducing the number of traffic crashes [20]. Another example is when the pedestrian is informed by a smartphone of an upcoming threat [19].

To ensure effective vehicle communication between pedestrians and other road infrastructure, additional devices like smartphones or tablets are employed to collect real-time data from multiple sources [21]. This type of communication is referred to as vehicle-to-device (V2D) connectivity and is commonly implemented via Bluetooth. There is a large number of connected devices, e.g., with long battery life, on the Internet of Things (IoT) (or Internet of Vehicles (IoV)) and V2D applications, where transmission of low-volume data with low latency is implemented [22].

Guaranteeing continuous data transfer in IoT applications and all V2X communication management systems and network technologies is the priority. Such a case is referred to as vehicle-to-network (V2N) connectivity. For instance, all alerts regarding road and weather conditions from different points on a long route are transferred to the vehicle in advance, or communication with nearby vehicles via a cellular network is implemented using networking [4]. Together with networking, cloud computing (vehicle-to-cloud (V2C)) and data centers for vehicular applications are implemented as data management facilities [23]. Various software updates, remote vehicle diagnostics, and complex computations like machine learning tasks are commonly executed on the cloud [24]. It has been found that machine learning algorithms are effective enhancements of V2X systems and are capable of computing various complex statistical and prediction problems [25].

One of the main concerns to make autonomous driving available for everyone around the globe is the regulation differences between different countries or continents in terms of used frequency bands or the preference for specific communication technologies over others [26]. For instance, Long range (LoRa) and ZigBee operate on the frequencies of 433 MHz in Australia, 915 MHz in America, and 868 MHz in Europe [27]. Analogously, Dedicated Short-range Communication (DSRC) operates on the frequency bands of 902–928 MHz in America, 5.795–5.815 GHz in Europe, and 5.770–5.850 GHz in Japan [26].

A set of various local restrictions, achievements, legal regulations, and habits require detailed analysis and systematization for the further development of ITSs. This is true especially in terms of technological advancements in environment sensing, fast and efficient data processing, and the use of artificial intelligence (AI) and signal transfer.

The motivation of this review is to systematically evaluate and, in a concentrated manner, present the latest V2X-related research and information which directly relates to the data types and methods used for ensuring reliable efficient and secure communication. Communication complexity strongly depends on the automation level, as a variety of data and tasks increase significantly. This information is relevant for further experimental research on data transferring in autonomous vehicular networking. This review focuses on the information, data types, communication equipment, tools, and machine learning methods used to process and optimize the collected data and the communication technologies used to transfer the data.

## 2. Method of the Selection Process

The search method for this research was based on [28]. Different databases such as MDPI, IEEE Xplore, Science Direct, and Google Scholar have been utilized, and some others were also explored because of several limitations (e.g., the article is only accessed in specific databases) after analyzing the reference lists. Several criteria (specifically for V2X communication) for articles have been defined for inclusion in this survey, as follows:

- Is focused on sensor applications;
- Is focused on equipment utilization;
- Is focused on machine learning adaptations;
- Is focused on data exchange;
- Is focused mostly on V2V and V2I connectivity.

Correspondingly, defined exclusion criteria are as follows:

- Articles older than 5 years are excluded with some exceptions after reviewing reference lists;
- Articles not specifically focusing on vehicular communication or data gathering were not selected;
- Articles focusing on railways, sea, air, or military transport were discarded.

**Figure 2:**
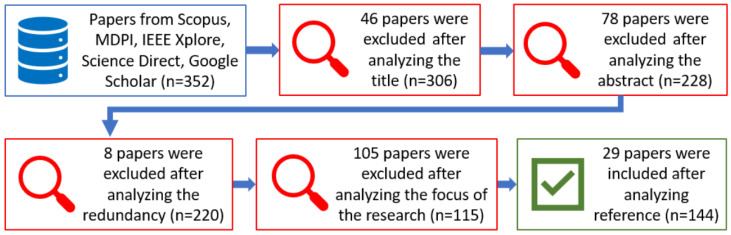
A systematic review process of the literature.

The selection approach for this manuscript was implemented by using V2X-related keywords, such as “car2car”, “vehicle2vehicle”, “vehicle communication”, “car2infrastructure”, “vehicle2infrastructure”, “v2x communication”, “smart cars”, “vehicle network”, “smart parking”, “road signs”, “traffic signs”, “vehicle detection”, and “vehicle sensors”. The complete simplified selection procedure is shown in Figure 2.

The search procedure gave an extensive result list, but the authors used only verified and rectified papers.

## 3. Technologies in Vehicles-to-Everything Communication

### 3.1. Sensors

**Figure 3:**
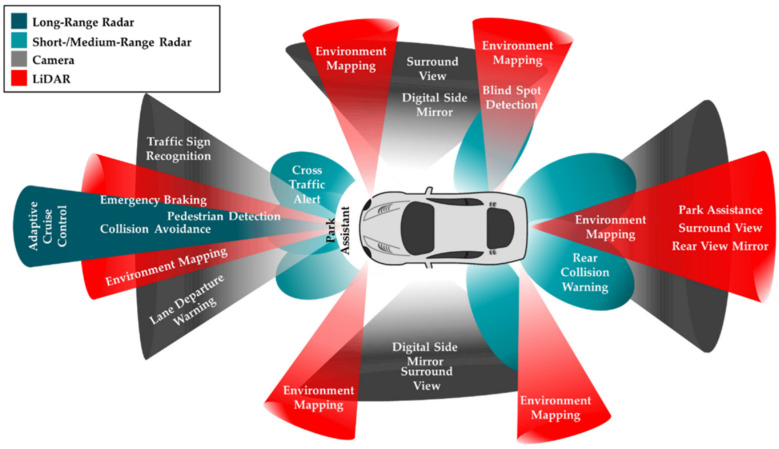
Vehicle environment sensing [29].

According to [29], sensors used in V2X communication can be classified into two groups: internal and environmental. Internal sensors measure such parameters as the vehicle’s motion, dynamic state, wheel speeds, and braking acceleration. Typical examples of internal sensors are accelerometers and gyroscopes. Environmental sensors monitor external objects like road signs and pedestrians. Typically for such applications, various cameras and radar-based sensors are used. In terms of sensing technologies, the evaluation of internal parameters is more developed and relies on older, reliable methods and technologies tested in various practical applications; contrary to environment sensing (Figure 3), there still exist many uncertainties requiring comprehensive research and validation.

In V2X communication, sensors installed in the infrastructure also play an essential role. For example, proximity sensors [30] could be implemented in the infrastructure to monitor the absence of vehicles for effective localization in parking lots or enroute. Radio-Frequency Identification (RFID) tags [31] could provide relevant information about various objects (road signs, etc.).

For of a large variety of measured quantities, sensor classification based on their operating principles allows for representing the current situation in the research area and reveals gaps for future development. Therefore, further tables (Table 2 and Table 3) present different types of sensors and their uses in V2X.

Depth (ToF), RGB, and RGB-D cameras are mostly mounted at the position of the front window and/or the rear window in vehicles [41]. Calibration is needed to avoid the distortion of images and for applications requiring data fusion (e.g., with LiDAR measurements) [42]. In ML, data from cameras are used to train ML models, e.g., object classification [43]. Another type of camera with a CMOS image sensor exploits the rolling-shutter effect—a picture is captured line by line from top to bottom [39]. On-road area cameras like CCTVs [47] and IPs [48] are built for vehicle observation.

**Table 3:** Proprioceptive sensors used in V2X.

Sensor	Measurements	Advantages	Disadvantages	Refs.
Magnetic sensors, magnetometer	Magnetic field or magnetic dipole moment	It has high sensitivity, small size, flexible installation, and strong anti-interference ability	Not susceptible to adjacent vehicles and can be affected by the magnetic signal dead zones	[15,49,50,51,52]
Accelerometer	X, Y, and Z-axis acceleration data along with latitude and longitude data	Portable, high-frequency, simple interface	Sensitive to external vibration and noise	[53,54,55]

Proprioceptive sensors detect the state of a system. The information from magnetic sensors and magnetometers covers the orientation estimation in combination with other on-board inertial measurement units, e.g., accelerometers and gyroscopes [50]. Sensing data from smartphones and ML algorithms are used to detect vehicle user status, i.e., inside or outside the vehicle, while parking occupancy is detected via a combination of infrared detectors, and distance sensors [52]. The accelerometer is one of the widely used sensors that can be a separate devices or embedded into a smartphone. It is used as an internal positioning system (IPS) as a motion and orientation sensor along with gyroscopes, GPS, and digital compasses for mapping movements (outside the vehicle or by driving it) of the user for short distances. It is also detects vehicle abnormalities, such as those caused by vibrations, e.g., loosening of wheel fixing bolts before riding or speed bump detection [53].

**Table 4:** Exteroceptive sensors used in V2X.

Sensor	Measurements	Advantages	Disadvantages	Refs.
LiDAR sensor	Position/distance, angle, and velocity measurements of a specific object	The distance to an object and the accuracy are significantly higher than from a radar, high angular resolution, good mid-range detection	High computational resources, uncertainty of data interpretation and analysis	[32,56,57]
Radar sensor	Position/distance, angle, and velocity measurements of a specific object	Can work in bad weather conditions	Have difficulty providing high-accuracy readings	[58]
Infrared (IR) sensor	Distance estimated by reflected IR light from the object’s surface	Can measure large distances and in a wide area	Difficult to distinguish the color or object from the complex environment, quality can be improved by using Gamma Correction method	[9,59]
Light Dependable Resistor (LDR) sensor	Measures light intensity mostly identifying if a light is present or not	Simple to use and integrate	The height of the vehicle has a severe impact on the accuracy	[30]
Ultrasound/ultrasonic sensors	A vehicle in smart parking lots or a vacant lot	Low cost and required simple installation	More suitable for outdoor environments, sensitive to temperature changes and extreme air turbulence, limited range	[51,60,61,62,63]
Acoustic sensors	The presence detection of an object	No needed a direct line-of-sight	Struggle to determine object sizes	[64]
Radio-Frequency Identification (RFID)	Coded readings	Fast and accurate identification, programable configurations of keys	Additional infrastructure is needed	[63,65,66,67,68]

Exteroceptive sensors measure the state of an environment. Examples are shown in Table 4. Radar sensors are found to be used in combination with other on-board sensors, e.g., cameras, LiDAR, and odometer measurements to obtain information about the surrounding environment. The radar sensor commonly is located on the front of the vehicle [58]. Additionally, LiDARs are used mostly in combination with other on-board sensors, e.g., cameras, radar measurements, and GNSSs (Global Navigation System Satellites) for C-V2X wireless technology. A LiDAR-based image processing approach is used with ML methods. These can generate a precise 3D (point cloud) map of the surroundings [32]. RFID technology and FMCW radars (or mm-wave radar) can also be used to locate the tags [29].

**Figure 4:**
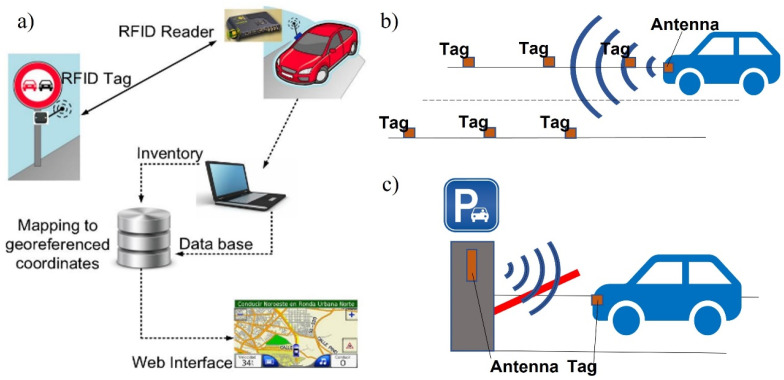
RFID usage possibilities (**a**) road signs; (**b**) tunnels; and (**c**) parking places [31].

From the IR sensor, the gray map is in front of the vehicle, and according to it, the tracking is judged. As an example, according to sensor measurements between other vehicles, vehicle velocity can be adjusted [59]. LDR sensors are quite often used with vehicles in smart parking systems and are based on the shadow detection method [33]. Ultrasound/ultrasonic sensors help to identify if the vehicle is in a smart parking lot or a vacant lot [63]. Large, high-density networks of parked vehicles can be recognized more easilyusing RFID technology compared to cameras. Only an RFID tag with a unique identification code needs to be installed within the vehicles or road signs to be read [68]. Mostly, RFID tags are used for vehicular use, e.g., parking places (Figure 4a) or tunnels (Figure 4b), where network technologies are weak, or for the road sign (Figure 4c), e.g., behind obstacles, during bad weather conditions, or at night for enhanced localization and recognition. They are also used for security authentication.

Also, it should be mentioned that in terms of V2X communication, due to the implementation of multiple sensors based on various physical principles, sometimes sensors are classified according to the operating range, communication technology, or implementation method. The most typical cases are summarized in Table 5.

As mentioned before vehicular communication systems consist of various V2X elements, and it is necessary to evaluate sensors properties and functionalities to choose accordingly for the required task. Depending on the specific task, properties like accuracy, measurement range, robustness, and cost must be evaluated. Also, functionality is a very important factor. For example, V2V and V2P elements require a more local detection approach, and sensors like LiDAR, ultrasonic, and infrared should be taken into account. On the other hand, V2I elements are used to communicate with the infrastructure, and RFID or camera devices should be taken into consideration for integrating elements not only into the vehicle but into the infrastructure itself. This can also allow for the extension of vehicular communication systems outside traffic, for example for parking place monitoring.

### 3.2. Communication Equipment and Tools

Additional equipment and tools are necessary in V2X communication that interacts with surroundings by indicating the action, e.g., break status indication via LEDs, storing measured or other important information on cloud servers, or preprocessing and fusing measured data using additional fillers. This technology also requires communication with satellites to monitor essential navigation data. These are several examples; more are presented in further tables (Table 6, Table 7 and Table 8).

Reliable data flow and storage are essential for vehicular communication systems. Because of the listed advantages and disadvantages presented in Table 6, each communication equipment has its place in the overall functionality of the urban system. This equipment enables communication between all elements of vehicular communication systems.

Filters are essential tools to deal with the raw data of the sensors, which, after postprocessing, can be manipulated much more efficiently. Some key factors to take into account when choosing a filter for a specific task are computational load, ability to deal with non-linear data, and noise. As data can vary depending on their physical nature, the task and functionality of the device’s AI machine learning methods are commonly integrated which will be introduced in the next chapter.

Because of the wide variety of devices used in vehicular systems, specific units are commonly used to interconnect different sensors or communication devices. Modules make it easier to set up and integrate required devices. Also, as shown in the table above, these units serve the purpose of classifying devices according to their applications, making it more convenient for vehicular system development.

### 3.3. Machine Learning Tools

Various artificial intelligence (AI) and specifically machine learning (ML) algorithms are used to enhance the quality of different vehicle communication systems. All ML algorithms can be separated into four distinctive categories: supervised, unsupervised, semi-supervised, and reinforcement learning [49]. In V2X communication, all four types of ML are applied. Supervised learning could be used to detect the occupancy of a parking lot by using labeled data to solve classification and regression tasks [95]. Unsupervised learning is more suitable for data grouping (clustering) tasks and could be used to group various types of vehicles according to their shape [82] or similar tasks. Semi-supervised learning is used when there is a lot of unlabeled data, but in combination with a small amount of similar labeled data, the pattern can be trained and used for classification tasks [96]. Reinforcement learning provides the most positive outcome in a sequence of decisions by ignoring irrelevant information during the training [97]. For example, it can be used to define the best vehicle acceleration at any point of the route to minimize fuel consumption.

With the development of microcomputers and the increase in computational power in edge computing devices, ML implementation in V2X communication has become possible. Research performed by W. Tong et al. [25] pointed out that V2X and ITS systems, together with AI can expand the driving perception and predict potential accidents to avoid them, enhancing the comfort, safety, and efficiency of driving. They can also enable real-time traffic flow prediction and management, location-based applications, congestion control, and enhanced capabilities of self-driving vehicles [98]. The authors of [99] proposed an idea to use AI to identify and monitor the authorized drivers and their state, count the number of passengers in the vehicle, and detect an unattended child in a vehicle, thus increasing the safety inside the vehicle. In contrast, research presented in [100] was focused on vehicle safety. It has been revealed that AI algorithms can overcome some important challenges for V2X communication security systems. AI algorithms could minimize delays caused by security key distribution for authentication and optimize data fusion procedures in terms of time and computational resources.

One of the most researched and technologically fulfilled ML implementations in V2X communication is vehicle localization. A summary of the research reported in this field in the last 5 years is presented in Table 9.

Another main aspect of the training is the material used to train ML models or architectures. Most datasets are open source and can be composed of different information specifically for any ITS field of interest, which mostly consists of numerical, statistical, or visual information. As an example, images of different road signs [106], statistical historical data of parking lot occupancy [107], and relevant sensor data [54] can be found in datasets. More summarized datasets of vehicle-related detection can be found in [108] with more comprehensive information including environmental information, sensors used for the data collection, format, and capacity.

**Figure 5:**
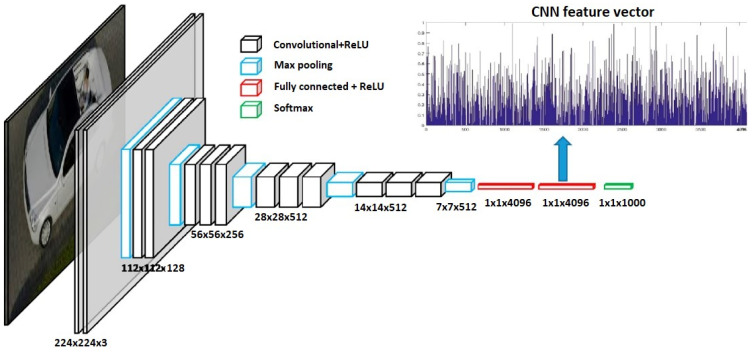
Example of CNN adaptability for vehicle recognition [38].

From Table 9 it can be noted that CNN-based ML architectures are used the most for object recognition and localization since images are used. In Figure 5, the example of CNN architecture when a bunch of images of different vehicles are used to recognize the absence of a vehicle is presented from [38]. As can be seen from these data, different CNN architectures can be built. For instance, in Figure 5, the network is composed of 13 convolutional layers, 5 pooling layers, and 3 fully connected layers, where ReLU is an activation function, and at the end, there is another activation function–Softmax. The key point before using this network is that the images have to be resized to the same dimension first.

An interesting point of view has been provided in the research [109], where several aspects of CNN architecture that also can apply to other ML architectures have been exposed:

- With more available data, more reliable classification results could be explored;
- If a network is calibrated well enough–it is not necessary to update the calibration on new data;
- Increasing the number of different drivers with different driving performances will decrease the performance of classification, and thus more data are required to receive similar classification results;
- Aadditional information, e.g., road or weather conditions and vehicle type, could affect the overall performance of classification;
- One of the limitations of CNN is that the input must be in the same dimension, whereas recurrent neural networks and long short-term memory networks are more flexible regarding the input dimensions.

In Table 10, machine learning methods are presented specifically for perception and best selection outcome, where CNNs are widely used, as well as some forms of YOLO (You Only Look Once) model variations [110]. These methods focus more on monitoring parking infrastructure and vehicles to predict the most optimal options for space availability.

Without these applications, ML is also used to optimize the performance of autonomous driving. Some of the examples are presented in Table 11.

Many other applications of ML were applied in recent research oriented toward some outcome prediction and other traffic parts recognition systems. Some of the examples are presented in Table 12.

As shown in Table 10 and Table 11 many different ML architectures are applied, enhancing the performance of regular search algorithms which would otherwise consume a lot of time dealing with monitoring parking spaces, recognizing road abnormalities, and other traffic features as mentioned before. Nevertheless, a lot of detailed information must be extracted from everyday traffic scenarios and simulated or classified using other methods to prepare suitable test input data and labels for ML algorithms.

### 3.4. Communication Technologies

A vehicular ad-hoc network (VANET) is a variation of the ad hoc network and mobile ad hoc networks (MANETs), where nodes (i.e., vehicles, and internal sensors) are communicating mostly wirelessly and only between each other [123]. It can be easier to implement because no infrastructure, like RSUs, is needed to be used as the central server, thus increasing the communication efficiency and road safety in intelligent transportation systems (ITSs) [20]. As an example, [75] has provided some cases of VANET in autonomous smart parking, like real-time occupancy monitoring of the parking lot, whilest [124] has presented the VANET-based architecture, which covers security services, network, and link layers and thus provides improved computations for collision probability and preventive measures for cooperative collision avoidance. One of the medium-range communication technologies, the Dedicated Short-Range Communication (DSRC), has been approved and considered by the CAR2CAR Consortium for common use for VANET [125]. Other communication technologies based on transmission range used in VANET and ITS applications can be divided into short- (<100 m), medium- (~100 m), and long-range (>10 km) communications [3]. Descriptions of each communication technology are provided in Table 13, Table 14 and Table 15 for short-, medium-, and long-range communications respectively. It can also be noted that different communication technologies are commonly defined by different standards such as 3GPP (3rd Generation Partnership Project) [126] and IEEE 802 (Institute of Electrical and Electronics Engineers) [127] with collections of networking technologies such as Ethernet and wireless.

Bluetooth is periodically used in close proximity as a short-range communication method [115]. Furthermore, compared to Bluetooth, BLE (Bluetooth Low Energy) applications are similar; however, it is a more energy-efficient technology. It works with a low transition range [15]. Using UWB, devices can operate at low power using short pulses of 3.1–10.6 GHz. Signals can penetrate through construction materials except metal surfaces [76]. Visible Light Communication or VLC transmits wireless Internet data at very high speeds using only light beams and can reach up to 100 Gbps. However, the modulation needs to be reliable under high vehicle density scenarios and variable road environments [39]. In addition, beaconing communication has low transmission power and a low-frequency band of approximately 10–50 Hz [123]. ZigBee technology consumes less energy compared to Wi-Fi and Blue-tooth and it is inexpensive to implement [65]. Compared to other short-range communication technologies, ZigBee can be used in a range of up to 100 m (sometimes it is considered as a medium-range communication as well). It is less sensitive to noises and obstructions by vehicles in terms of the bit error rate (BER) and signal-to-noise ratio (SNR) [128].

For more information about short-range communication technologies see Table 13.

**Table 13:** Short-range communication technologies used in V2X.

Technology	Range	Applications	Refs.
Bluetooth (IEEE 802.15.1)	Up to 10 m	Commonly used for the user to access devices and notifications, e.g., in terms of a parking lot’s availability via smartphones or tablets; is used for inner communication between OBUs in the vehicle, e.g., for notification about an engine problem	[77,129]
BLE (IEEE 802.15.1)	Up to 5 m	Commonly used for notifications and with battery-functioned small devices.; a small amount of data is used for transmission, e.g., only the device ID; not suitable for inter-vehicular communication or precise localization applications because of its severe fading effects	[15]
UWB (IEEE 802.15.3)	Up to 10 m	Because of low signal amplitude, it is less sensitive to the noisy environment and thus has more secure signal transmission, e.g., secure locking and unlocking of vehicles using key fobs; uses radio-based localization with the accuracy of sub-meters	[37,76]
Visible Light Communication (VLC) (IEEE 802.15.7)	Up to 6 m	Data transmission between two adjacent vehicles, although a stable communication link between the two vehicles is needed and since the distance between the transmitter and the receiver increases, the transmitted power must also increase; drawbacks can be eliminated by applying distant measurement sensors, e.g., LiDAR additional optical systems to boost the received power	[34,39]
Beaconing	Up to 5 m	Suitable for small amounts of safe data transmission and can be easily implementable; this type is used as an auxiliary means for other technologies, e.g., BLE, to periodically transmit data in the form of beacons with adjustable rate	[7,123]
ZigBee (IEEE 802.15.4)	Up to 100 m	One of the common communication choices in smart parking systems; it can broadcast small amounts of data over a short range with a smaller energy consumption compared to Wi-Fi and, theoretically, up to 65 000 devices in a network can be managed; this technology finds it more difficult to penetrate obstacles compared to Wi-Fi, although this drawback may apply to tunnel communication	[65,71,128]

Going into the medium range, Dedicated Short Range Communication (DSRC) technology is commercially available and the (WLAN) IEEE 802.11p protocol emerged as the first ever standard for V2X communications, which does not require a basic serves set [130]. However, the 11p protocol still lacks safety for critical communication and autonomous driving. For this reason, the IEEE 802.11bd protocol is being used for new generation V2X developments to improve these shortcomings. Some of the safety requirements are to be reached at 99.99%, including a latency of no greater than 3 ms, making it very challenging. Current DSRC systems based on the 11p protocol have a latency of about 100 ms, as long as traffic is not too dense [131]. Systems incorporating DSRC are also highly expensive. In comparison, Wi-Fi can provide a stable performance in terms of reliability and latency [125]. On the other hand, Wi-Fi communication can operate under the circumstances of obstructions, with the help of multipath propagation [132]. The main drawbacks are its latency and short range, which can be overcome using other technologies, e.g., DSRC or LTE [133]. Further details are provided in Table 14.

**Table 14:** Medium-range communication technologies used in V2X.

Technology	Range	Applications	Refs.
DSRC (IEEE 802.11p/ IEEE 802.11bd)	Up to 1 km	Used in Vehicle Safety Communication (VSC) in urban environments because of its robustness against severe fading in highly vehicular infrastructural environments	[36,94,125]
Wi-Fi (IEEE 802.11ac/ IEEE 802.11ax)	Up to 100 m	One of the common connections between vehicle and driver or passenger on-road or in the parking lot, e.g., via smartphone, and to transfer related information to/from the database	[16,92,132,133]

Compared to Wi-Fi (54 Mbps), Wireless Intero-perability for Microwave Access (WiMAX) can provide higher Internet access speed (up to 70 Mbps). It is expensive to install and operate because a line of sight (LOS) is needed, and it also has higher latency [134]. The 4G Long-Term Evolution (LTE) V2X has high throughput, low latency (10–100 ms), and is one of the main short-range communications [2]. It has two physical channels one for the data carrying and the other for the control of information for decoding the data carrying channel [132]. Although, to meet the requirements of 3GPP, the effective use of network resources is needed and 5G (LTE) provides an even higher speed and a latency as low as 35 ms. In reference [135], the effective edge nodes resource allocation method is proposed by processing the demanded user data using the centralized RMU algorithm in the core network. The 5G New Radio (NR) is one of the latest technologies for which 5G infrastructure first needs to be deployed and adopted. There are two communication modes: one for direct vehicular communications via the UU air interface under the coverage of the cellular network, and another for the out-of-coverage area of the cellular network via the PC5 interface [126]. Currently under development, 6G-V2X communication technology is the latest technology in the THz band and can support even better hyper-fast, ultra-reliable, and low-latency communication compared to 5G-NR [136]. Cellular V2X (C-V2X) consists of 4G-LTE or 5G-NR communication technologies and thus can cost-efficiently provide a longer range than Wi-Fi with the extended detection of the coverage and blind spots [26]. Long-Range (LoRa) communication technology has a data transmission rate of 300 bps –37.5 kbps and is low-power. Data transmission is reliable with low latency; however, the transmission throughput is very low as well [27]. The last of the long-range communication technologies is Narrowband (NB) IoT, which has low power consumption, high performance, high security, and wide area coverage communication, and signals are sufficient to penetrate through obstructions [137]. More details can be found in Table 14.

**Table 15:** Long-range communication technologies used in V2X.

Technology	Range	Applications	Refs.
WiMAX (IEEE 802.16)	Up to 50 km	WiMAX is mainly considered as a supplement to Wi-Fi	[134]
5G NR (3GPP Rel. 16,17)	Up to 5 km	High throughput with very low latency (1 ms); can provide higher data rates and be ultra-reliable for critical applications, e.g., secure and efficient control functions in autonomous vehicles; additional research is being performed to evaluate the support and enhancement capabilities of this communication	[126]
C-V2X (3GPP Rel. 14)	Up to 1 km	Featured with low latency and high reliability. ensuring critical and safe vehicle sensors connectivity; used for keeping safe distance and speed between vehicles, connection with roadside infrastructure, sensing of other roadside participants; can overcome LOS issues with long-range perception using cellular networks	[26]
LoRa (Based on IEEE 802.15.4)	Up to 20 km	According to research, LoRa can cope with real-world scenarios with actual vehicles at higher speeds and a dynamic environment in terms of reliability and performance	[27]
6G-V2X (under development)	-	Massive information exchange technology by combining several communication networks, e.g., satellite and unmanned-aerial-vehicle (UAV) networks, with a combination of ML methods	[136]
4G LTE V2X (3GPP Rel. 14, 15)	Up to 20 km	Can be used as an alternative to Wi-Fi; 4G LTE does not support C-V2X applications as well 5G NR, although the infrastructure is more deployed	[2,132]
NB IoT (3GPP Rel. 13)	Up to 10 km	Suitable in smart parking systems; small data volume amounts are transmitted, e.g., parking lot availability and parking time	[137]

One of the main concerns in V2X communication research that can be found in the literature is the system performance in terms of the bit error rate (BER), signal-to-noise Ratio (SNR), signal throughput, and latency [123,129]. BER is defined as the ratio between the number of unsuccessfully transmitted bits and the number of all transmitted bits. In VLC communication, it can be optimized by having a lower pulse width ratio or adjusting other settings of the modulation [34,39]. For instance, ref. [123] stated that in order to keep the BER lower, the received power at the vehicle must be larger or equal to the receiver sensitivity. The researches of [129] conducted studies on the BER and throughput analyses for the performance of Bluetooth. Some 3GPP standard communications [126] have used Long-Term Evolution (LTE) turbo coding to minimize BER and found that increasing vehicle density does not affect it. In the same research, different SNR values were considered for several scenarios. The researches of Ref. [1] evaluated C-ITS architecture based on millimeter-wave (mmW) and Free-Space Optics (FSO) technologies in terms of SNR.

In another example, ref. [22] simulated the systems throughput and the transmission latency with different vehicle densities in the rural and the urban scenarios in the 5G network environment, which is based on Software-Defined Networking (SDN). Road weather and traffic influence on throughput, packet loss, and latency have been evaluated in [12], comparing LTE and the 5G Test Network (5GTN). Here 5GTN showed better results compared to LTE. Signal latency and throughput have been used to evaluate the smart parking network in [61] to optimize the placement of RFID-based WSNs. In [100] information interchange latency between vehicles and RSUs was significantly decreased when security keys and a key-sharing network for signal security were used with the help of ML algorithms.

Software-defined radio (SDR) testbeds can be used to evaluate the broadcast distance power, packet delivery ratio, throughput, latency, reliability, and packet loss rate of the signal, e.g., to reduce the stopping distance [125]. It also can be used to analyze the parameters and identification of RFID tags [138]. SDR can provide functions such as signal modulation and demodulation, spectrum analysis and monitoring, filtering, and frequency selection and it is an open source.

V2V and V2I interactions can be implementable by applying different simulation frameworks. Different open source frameworks and platforms, which have libraries of different implemented models by the scientific community, can be found as Objective Modular Network Testbeds in C++ (OMNet++), Vehicles in Network Simulation (VEINS), Internet networking (INET) [139], and Simulations of Urban Mobility (SUMOs) [67]. These platforms are used to simulate real-life scenarios by determining a different number of variables of the V2X system, e.g., vehicle nodes, infrastructure nodes, and other specific nodes [118].

Furthermore, computational analysis using 3D ray-tracing tools and 3D ray-launching algorithms for V2I of WSNs can be simulated to evaluate the received power, power delay profile, delay spread, and coherence bandwidth [74]. These tools are used to model and simulate real-life scenarios for the deployment of radio planning and propagation monitoring in V2I environments representing terrain, buildings, pedestrians, vehicles, streets, and other geographic data, different frequencies, the height of the transmitter and receiver antennas, and transmission power. As an example, [73] used these simulations for the optimal distribution of WSNs and deployment of urban RSUs with affordable computational cost. The authors of Ref. [71] used it to utilize the deployment of WSNs in a tunnel scenario as a complex and singular environment considering limited dimensions and metallic elements within it, e.g., user pathways or service trays.

**Figure 6:**
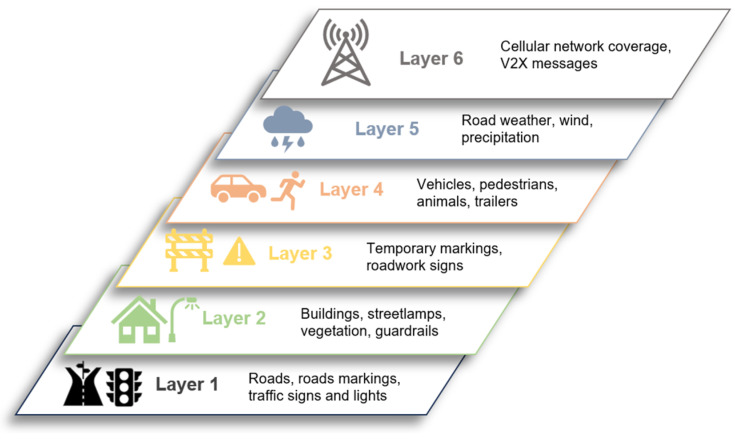
Six-layer map for V2X [140].

In order to facilitate the efficient navigation of digital maps, ref. [140] proposed a six-layer map model, designated to describe unstructured real-world operational design domains, as illustrated in Figure 6. Each layer contains distinct types of data, which are dedicated to a specific navigation task.

In order to utilize a six-layer map for V2X modeling in connected and autonomous vehicles (CAVs), it is essential to comprehend the contribution of each layer to the overall functionality and the manner in which the information within each layer is updated. This map encompasses the road network, roadside structures, modifications, dynamic objects, environmental conditions, and digital information layers. Each of these layers plays a pivotal role in ensuring the efficient and safe operation of CAVs.

Further tables (Table 16, Table 17 and Table 18) summarize different vehicle-related, infrastructure-related, and other data and their purpose in the V2X communication-related research, and are tabulated respectively.

Considering there will be more and more vehicles, especially electric vehicles, in the future, there is a strong likelihood that there will not be enough resources to charge vehicles fully or efficiently without the addition of more public charging grids (V2G). Therefore, more attention is needed to individualize smart charging systems at homes, housing estates, and apartment buildings (V2H) by utilizing previous research on smart parking systems.

The current challenge for most communication types and data transfer is to make them secure and robust. The more information is used in communication, the more it is responsible for different aspects of autonomous driving and the loss or overwriting of one part of the data sequence can vitally affect the whole system. Therefore, signal authentication in V2X communication is one of the main challenges.

## 4. Discussion and Conclusions

Sensors are an essential part of vehicular-to-everything communication, allowing for localization of vehicles, obstacles, and infrastructure elements like signs, traffic lights, road markings, etc. Integrating various sensors not only in vehicles but also in infrastructure elements allows for to the functional expansion of parking and tunnel monitoring, and thus better management of overall traffic and greenhouse gas emissions.

Vehicular communication systems require various sensors of different physical natures with essential properties like range, computational resources, robustness, and sensitivity to noise, which must be evaluated and then chosen according to the task. Autonomous systems have expanded the range of measurements, requiring us to take into account not only common features like speed and distance but also color and shape. This requires a synergy of sensors that can be achieved with the development of sensor fusion and ML methods and architectures.

Vehicular communication systems’ functionalities are very dependent on reliable data transfer. Communication can be influenced by many factors, including security, data corruption, bandwidth limitations, and physical interferences. Summarizing the review, three main scenarios of data transfer can be distinguished:

- Regular scenario. Data are transferred using OBU in vehicles (V2V scenario) using RSUs (V2I scenario) without significant or critical signal losses. However, the main challenge in adapting this equipment is the high speed of vehicles and the dynamic environment for real-time data transmission between vehicles or another RSU. Communication tools can be radio TX and RX, action TX, and sensor RX (e.g., light signaling and detection).
- Lack of data scenario. The system does not receive required data to properly complete the tasks, for example to follow road lines or to keep a constant distance from the car in the front. Data transfer is weakened by car overcrowding, dense infrastructure, or reflectors, e.g., buildings and power lines, low signal interruption, and hazardous environments affected by weather (blizzards, sand dust). Visual data can be affected by unwanted obstacles, (trees or bushes grown up in front of road signs which partially or fully block a view). A Software Defined Radio (SDR) testbed can be used to evaluate the broadcast distance power, packet delivery ratio, throughput, latency, reliability, and packet loss rate of the signal. Furthermore, computational analysis using 3D ray-tracing tools/3D ray-launching algorithms for the vehicle to the infrastructure of wireless sensor networks (WSNs) can be simulated to evaluate the received power, power delay profile, delay spread, and coherence bandwidth.
- Faulty scenario A common scenario has faulty information interpreted as correct data. For example, non-intentional road patterns may be interpreted as road lines. Faulty or unstable information is sent via V2V or V2I connectivity, and may be affected by high signal interruption, the risk of a hacked signal and being replaced by another, delayed signals, disappearing signals (tunnels, underground parking), or a misdirection situation of crossing cars signaling from the wrong direction (visual-based problem). Therefore, additional security key protocols (and time stamps) are necessary, and additional short-range communication infrastructure and additional object or light recognition (via machine learning) tools are a plus.

Integration of AI machine learning methods is essential to enhance and ensure reliable performance for data processing, which is essential in every level of a vehicular communication system, including recognition of system elements, decision making, data transfer, and planning. However, robustness of the AI system strongly depends on training data and monitoring of the model in real-time because the system can encounter unexpected values and data distribution drift over time. The most commonly used ML architectures for vehicle-to-everything communication include NN, CNN, KNN, RNN, decision tree, and adapted GA in some cases. Nevertheless, future challenges require us to search for new solutions. The hybrid architectures of ML are designed for different types of vehicle communication techniques. For example, while CNNs are more efficient with spatial data like images, RNNs deal better with sequential data. Joining these two networks, the spatial distribution of traffic from images and the sequential features of traffic dynamics can bypass the limitations of both networks. Another addition for ML architectures are transformers, which have encoder–decoder architecture and were first introduced for syntax and semantics characterization and translation tasks. Now, they are used effectively for vision tasks and demonstrate better results than CNNs. Developing a combination of DNNs and transformer architectures enables efficient real-time task classification and a smoother operation in the highest automation modes.

Further research of the mentioned ML algorithms and their combinations is essential for the development of intelligent transportation systems as the infrastructure of smart cities will grow in the future. Going forward, there are also plans to focus on vehicle localization in closed environments like tunnels to enable local detection of static and dynamic obstacles and to ensure communication with the target destination due to the predicte increase in transport flow in the growing city. Autonomous driving facilities must conform to the rules for vehicle-to-vehicle and vehicles-to-infrastructure communication.

## Figures and Tables

**Table 1:** The differences between automation levels.

Automation Level	Description	Data	V2X Elements
Level 0 No automation, driver only	The driver performs all driving tasks.	Manual control, no data transfer.	-
Level 1 Specific automation, driver is assisted	The driver performs most driving tasks but some vehicle functions can be assisted by the equipment.	Speed monitoring and control.	V2D
Level 2 Partial automation, driver is assisted	The driver performs fewer driving tasks but must be engaged since some functions like acceleration or steering are automated.	Steering and acceleration control.	V2V, V2I, V2P, V2D
Level 3 Self-Driving automation, partial driver interaction	The driver is only necessary to take control of the vehicle with notice, but not required to observe the environment.	Environmental perception of RFID tags, obstacles.	V2V, V2I, V2P, V2D, V2N
Level 4 High automation, specific driver interaction	The driver is not needed for autonomous driving to perform driving functions. The driver can take control of crucial driving tasks or in other specific circumstances.	Autonomous path following according to scanned road pattern data, tags, transmitting devices.	All
Level 5 Full automation, no driver interaction	The driver performs no driving tasks but can take control.	Interconnected data controlled with AI methods, connected to the Internet of things.	All

**Table 2:** Camera-based sensors used in V2X.

Sensor	Measurements	Advantages	Disadvantages	Refs.
Depth (ToF) RGB, RGB-D cameras	Different images are processed for color and depth recognition	Possible to interpret images for obstacle avoidance or movement tracking, land detection and tracking, license plate number recognition, etc.	High computational resources for feature recognition, less accurate distance measurement compared to LiDAR, and less range compared to radar	[32,33,34,35,36,37]
Camera with Complementary Metal-Oxide-Semiconductor (CMOS) image sensor	The modulated light source to decode the transmitted data	High resolution, low noise, high dynamic range, lower power consumption	Rolling shutter distortion	[38,39]
Closed-circuit television (CCTV) camera	Videos are used for a computer vision algorithm and detect vacant spots	Lower costs, can resist any weather constraints	Additional infrastructure required, might suffer from poor video quality	[40]
IP camera	Line-of-sight (LOS) and non-line-of-sight (NLOS) monitoring purposes	Can resist any weather constraints, better image quality than CCTV	Additional infrastructure required, higher cost	[41]
Neuromorphic camera (event-based camera)	Event base cameras are able to capture the change of pixel intensity as discrete events	Allows measurements with ultra-low latency, overcoming under sampling. Fast, real-time processing.	System can be overwhelmed if high-speed events are not intelligently processed and clustered	[42,43]
IR camera	Detects infrared light with a wavelength of 700–1000 and converts acquired heat values into corresponding color	Allows the detection of heat values for detecting obstacles in foggy environments or environments with limited visible light	Infrared radiation is reflected off glass, creating false detection scenarios	[44]
Fish-eye	Dome-like lens shape is used to gather light from different directions and a special mapping is used allowing to generate images with convex non-rectilinear appearance	Enables the observation of a wide field of view from shorter distances. Circular view coverage of surrounding area	Field curvature of fish-eye camera leads to astigmatism and higher-order chromatic aberration. Also suffers from optical artifacts	[45,46]
Cyclops camera	Cyclops is system which uses low-cost monocular cameras to perform physical identity binding between objects’ digital and physical identity	Enables the prediction of spatiotemporal traffic randomness, allowing for the identification of security attacks and the prediction of trajectories	System performance strongly depends on used cameras and target matching algorithms	[47]
Polarized camera	Camera has integrated polarization system, which allows it to acquire the orientation of the light oscillation that appears from reflected surfaces.	Allows the enhancement of contrast and more accurately detect objects in crowded and packed environments	Polarization filter can be costly. Requires more light than normal and its angle to the sunlight needs to be adjusted for maximum effect.	[48]

**Table 5:** Common sensor equipment definitions used in V2X.

Definitions	Application	Commonly Used Sensors	Advantages	Disadvantages	Refs.
Long-Range Wide Area Network (LoRaWAN) sensors	Used for determining the use of smart parking as a network infrastructure of sensors	Radar sensors	Low power consumption, long-range protocol, and covers larger areas.	Limited to a line of sight, audio and video not supported	[49,69,70,71]
Cellular sensor	Used for determining different sensors that are embedded into mobile phones	Accelerometers, gyroscopes, GPSs, cameras, magnetometers	Portable device with a variety of sensors providing a convenient set of tools	The challenge of developing and integrating portable sensors	[11,72]
Wireless sensor networks (WSNs)	Used for determining the sensor nodes scattered throughout some kind of area, i.e., parking area or roadway; measurements are collected into a station base and sent by wireless technology	RFID or proximity sensors	Accurate and fast identification of global points in the working environment	The main challenge is optimal node placement, and this can be achieved by using a 3D ray-launching algorithm	[61,65,73,74]
Vehicular Sensor Network (VSN)	Used for connected and autonomous/smart vehicles are seen as nodes of a heterogeneous sensor network and these vehicles are the central elements of interest	Proximity, radars, and cameras	Significantly improves the local view of the working environment, enabling detection, tracking, and identification objects	Data interconnection from sensors of different physical natures, thus sensor fusion and ML methods are commonly used	[75]

**Table 6:** Common sensors equipment definitions used in V2X.

Definitions	Description	Application	Advantages	Disadvantages	Refs.
Radio-frequency (RF) transmitter/ receiver	Technology that emits and receives the radio/electromagnetic signal by antenna	DSRC, LTE, and LoRa implementation to read passive or active RFID tags, e.g., parking places and road signs	Can be used for long-range applications and can pass through obstructions to some degree	Sensitive to other present electronic devices. Limited RF spectrum	[50,62,67,76]
Global positioning system (GPS)	Conventional positioning system to help the user easily identify and monitor the vehicle location, heading direction, and route	The receiver with the antenna uses the satellite-based system to acquire data about velocity, position, and timing	It helps track paths, predict and avoid road congestions, and, with additional sensors, the road irregularities	Signals are weak or highly distorted in urban environments (tunnels, multi-layer bridges, and streets beside high buildings), unsuitable for precise vehicle localization in such areas	[55,77]
Differential global positioning system (DGPS)	Ground reference system is used to measure errors and provide more accurate satellite data for local users.	Receiver on vehicle acquires differentially corrected data about velocity, position, and timing	Enhances GPS precision, allowing us to achieve an accuracy of up to 1–3 cm	Atmospheric errors still exist. Reference stations have to be built. Correction data transmitting/receiving provides some latency	[78]
Point positioning real-time kinematics global positioning system (PPP RTK GPS)	Positioning system with a single receiver, which acquires satellite data from ground reference networks	PPP RTK receiver with an antenna uses the satellite- and ground reference network-based system allowing us to obtain more accurate data about velocity, position, and timing	Enhances GPS precision, allowing us to achieve an accuracy of up to 1 cm	Limited by satellite conditions and coverage. Also, affected by environmental obstacles. Ground reference networks are needed	[79,80]
Controller Area Network (CAN) bus	Message-based communication with each device without the host computer using two wires	It is considered to be the main solution for transferring information between different units on-board	It is fast and cost-effective compared to other communication protocols	Bandwidth limitations, cable length limitations, number of nodes limitation	[2,14]
Light transmitter (LED, incandescent light bulbs)	Also known as LiFi (Light Fidelity) technology	Transmission is encoded by varying the pulse width modulation at which a light turns OFF and ON to generate binary sequences	High-speed data transmission	Obstacles and other light sources interfere with communication	[34,81]
Cloud service	High-capacity data storage with additional computing resources	To receive and contain space-consuming data, provide specific data, and execute resources required for computations, e.g., ML training; wide and long-range communication	Flexible, reliable, and efficient way to store and manipulate data	Possible outages depending on services, vulnerability to cyber attacks	[23,24,40,82,83,84]

**Table 7:** Additional filters used in V2X.

Filter	Application in Vehicles	Advantages	Disadvantages	Refs.
Kalman filter	Used for stabilizing received signal strength indicator values which are obtained by sensors from dynamic environments (values always changing)	Simple and computationally efficient	Strongly depends on an accurate model and initial conditions, convergence to suboptimal solutions because of possible errors in model and assumptions	[33,37,58]
Unscented Kalman Filter	Estimation of velocity and slip angle of vehicle are implemented together with Machine learning methods such as Convolutional Neural Networks or Radial Basis Neural Networks	Better estimation results with non-linear system	Computational complexity	[85]
Interacting Multiple Models filter	The objective is to enhance the resilience of navigation and to mitigate the impact of fluctuations in system models and measurement noise models due to external factors	Better estimation results with noisy data	A selection of the number and type of filter models is a prerequisite.	[86]
First- and second-order divided difference filters	This approach allows the filter to be implemented without the need for any knowledge of the partial derivatives of the system dynamics and measurement equations.	Capable of solving numerical instability problems in noisy nonlinear time-series prediction	Has limited accuracy because these methods are based on an approximation	[87]
Particle filter	Achieves better precision in localizing the vehicle position based on noisy information	The ability to handle non-linear and non-Gaussian dynamic models allows for application of this filter to a wide range of applications	Computationally can be very expensive. Sensitive to previous distributions of the particles	[88,89]
Symmetric measurement equation (SME) filter	An effective solution for multiple target tracking and is suitable for a densely-spaced large number of targets (like vehicles)	Low computational demands	Suffers from strong non-linearities	[90]

**Table 8:** Common equipment definitions used in V2X.

Definition	Description	Equipment	Application	Refs.
Electronic Control Unit (ECU)	Embedded systems within modern vehicles communicate over networks to control different vehicle systems	Commonly it is connected through a CAN bus	To communicate to other ECUs, sensors, and actuators (e.g., on-board units)	[2]
Computing/ processing unit	A separate unit to perform the additional computational power required and complex tasks	Commonly it is connected to the ECU or cloud	The computational burden and bandwidth-demanding performance of AI and machine learning tasks	[91]
On-board unit (OBU)	The embedded vehicles system has necessary sensors and processing units to collect surrounding information and process to transfer it	Uses a wireless transceiver and receivers to communicate with surrounding vehicles and infrastructure	Transmission of location information (e.g., vehicle direction and speed) to the ECU	[19,27,92,93]
Road-side unit (RSU)	Surrounding infrastructures that are equipped with a processing unit and transceiver	Uses a wireless transceiver and receivers to communicate with surrounding vehicles and infrastructure	Used to warn and suggest a direction to the nearby vehicles depending on the received data by other vehicles	[10,27,73,74]
Cooperative intelligent transportation systems (C-ITS)	Transportation system with the enabled cooperation	Surrounding vehicles or RSUs as sub-systems	Data exchange between two or more ITS sub-systems enables and provides an enhanced service level with better quality	[1,94]

**Table 9:** Machine learning methods used for vehicle localization.

The Aim of Use	Approach	ML Method(-s)	Achievement	Refs.
Obstacle avoidance, localization, mapping, navigation	LiDAR-based image processing	CNN	Results show that the obstacle recognition rate can reach up to 97% at a far distance	[6]
Find areas where vehicles are located and their approx. number	In combination with CNN, using input images and obtained feature vectors after training	Support vector machine (SVM) and faster regions with CNN	The faster R-CNN method achieved better classification results compared to SVM	[24]
Brake light recognition from a single image in real-time	Brake Lights Patterns (BLP) database	AlexNet (CNN-based)	The ML model can classify vehicles when brakes are pressed or not pressed in real-time; the prediction accuracy is improved by using LiDAR sensor data fusion	[35]
To localize the car using an unmanned aerial vehicle (UAV)	Use of images taken from UAVs with a combination of linear SVM	Convolutional Neural Network (CNN also known as ConvNets)	The proposed method outperforms the vehicle’s catalog-based and feature extraction of histograms of gradient methods in accuracy and computational time	[38]
Multi-detection and tracking; recognition and deviation of different vehicles and other objects in various circumstances, including parking lot and number plates	Roboflow dataset of real-time video sequences of road traffic with Python OpenCV; videos taken with a CCTV camera to train ML models	YOLO-based models and combinations with other ML models	These systems are capable of identifying different traffic objects in various circumstances with accuracy in range from 80% to 96%, depending on the architecture	[82,101,102,103,104]
Vehicle detection in complicated environments and weather conditions	The KITTI (Karlsruhe Institute of Technology and Toyota Technological Institute) dataset	Single-shot multi-box detector (SSD) (CNN-based)	With an average mean accuracy value of 92.18% and an average processing time per frame of 15 ms; the proposed algorithm can achieve simultaneous accuracy and performance in real-time	[105]

**Table 10:** Machine learning methods are used for perception and best selection outcome.

The Aim of Use	Approach		ML Method(-s)	Achievement		Refs.
To sense the vehicle presence in the parking lot by using the beacon-based mechanism	Use of radar and magnetometer sensors and time-relevant data, trained with Keras library in Python		Neural networks (NN)	The accuracy of the NN approach reached up to 97%. The proposed approach reduces the costs of sensor production by at least half		[49]
To select the best parking place for an autonomous car in terms of the accessibility rate	Training has been performed using MATLAB with a real-life scenario composed of 300 places divided into five branches equally and VSN		Tree-based algorithm	Results are close to the optimum for the case of introducing one more autonomous vehicle and outperform the optimum method when successive vehicles are the parking		[75]
To classify car parks	Time series characteristics of car parking data		K-medoids clustering (K-means-based)	The model performs better clustering results compared to the dynamic time-warping model		[95]
To release parking lots that are open to specific groups for public usage in shared city parking	Use of real-time collected parking data, 168 inputs (hourly data for 7 days), and output prediction for the subsequent 168 periods		Recurrent neural network (RNN)	RNN model obtained the smallest testing error for artificial and actual datasets compared to other ML algorithms		[107]
To analyze high-frequency GPS location data of individual car drivers	Use of information about speeds, acceleration, deceleration, and direction changes		CNN	Developed a model capable of successfully performing classification tasks by allocating individual car-driving trips		[109]
To extract the main features underlying the time-series data in historical driving memory		The Next Generation Simulation of high-quality traffic datasets with real-world trajectory data and extracted main features of the time-series driving memory data	Auto-Encoder (NN-based)		Results show that velocity, relative velocity, instant perception time (IPT), and time gap are the most relevant parameters	[111]

**Table 11:** Optimization approaches of machine learning methods in V2X.

The Aim of Use	Approach	ML Method(-s)	Achievement	Refs.
To minimize the number of signals which can be used to recognize the activities performed while driving	With 20 drivers and the use of sensor data from three-point electrooculography (EOG) electrodes, three-axial accelerometer, and three-axial gyroscopes	One-dimensional convolutional neural network (1D CNN)	The ML model was able to classify the actions performed by drivers accurately, with the maximum accuracy of 95.6% on the validation set and 99.8% on a training set	[54]
To improve the detection and classification accuracy of various distant vehicles or other traffic objects	Use of the UA-DETRAC, traffic lights, and other photos of vehicle datasets for multi-object detection and tracking	YOLO-based models and combinations with other ML models	The evaluated classification of different kind of vehicles’ performance metrics i.e., accuracy, precision, and recall are raised.	[112,113,114]
To enhance the quality and safety of autonomous driving control	Use of the AirSim open-source simulator as the training data for real-time images of the road	Deep Reinforcement Learning (DRL)	Appropriate reward-generation method to improve the convergence speed of the adopted models and the control performance of moving driverless vehicles	[115]
To simplify the search for a motion representation	Use of three datasets of image information between frames: Something-Something V1, Something-Something V2, and Kinetics-400	Spatiotemporal motion network (SMNet) (CNN-based)	ML 2D CNN-based networks exceed other methods in motion recognition and do not require some pre-calculations, thus reducing computational costs and work time	[116]
To minimize the power grid load variance	Implemented and tested in MATLAB considering statistical information about the target load, the actual power grid load, and the capability of the grid-connected EV	Genetic algorithm (GA)	The proposed algorithm shows a better performance in percentage improvements of peak and valley load difference	[117]
To improve the support for V2X communications by finding optimal UAV positions	Considers the current flight altitude of UAVs, simulations have been executed by using OMNeT++, SUMO, and Veins tools	Particle Swarm Optimization (PSO) algorithm and GA	With the dynamic movement of the vehicle on the ground, and the existing flight restrictions, the best position of the UAV can be determined in real time	[118]

**Table 12:** Prediction uses of machine learning methods in V2X.

The Aim of Use	Approach	ML Method(-s)	Achievement	Refs.
For the detection of road abnormalities (e.g., speed bumps)	Use of collected accelerometer and gyro sensor data. The ML model has been trained by using the R programming language package GALGO	Tree-based algorithm	The achieved accuracy of the trained model was 97.14% during the test	[55]
To learn and predict when a fleet of vehicles was parked close to charging stations	Uses historical data from a fleet of 48 vehicles, including time and GPS location data Training is performed using Microsoft Azure and the Google Cloud platforms	Automated machine learning (AutoML)	AutoML achieved the best performance, with a prediction accuracy of 91.4% when individual vehicles could potentially connect to charging stations	[83]
Traffic sign detection and recognition with the consideration of the effects of by the environment	Uses the German Traffic Sign Recognition Benchmark (GTSRB) dataset to train the ML model in the Python library PyTorch	LeNet (CNN-based)	The accurate recognition rate of traffic signs reaches 99.75%, and the average processing time per frame is 5.4 ms; compared to other algorithms, the proposed algorithm has better accuracy, real-time performance, strong generalization, and efficiency	[106]
To detect a malfunctioning thermostat even if the car equipment does not indicate it	Use of collected accelerometer and gyro sensors’ real-time data from Hyundai i30 vehicle, which consist of 44 h of driving	Decision tree	The best accuracy of 88.9% was reached	[119]
To allocate the optimal minimum contention window for the vehicular node	Uses the replayed history data and obtained age dataset through real-time protocol simulation.	Deep-Q-learning	The model has a high degree of adaptability and can achieve a relatively high level of age equity benefit	[120]
To predict parking space availability	Uses the collected data composed of parking ID, timestamps, duration, and space status	Random forest, decision tree, and KNN	Algorithms separately outperform complex algorithms such as NN, in terms of higher prediction accuracy by providing comparable prediction results of available parking space	[121]
Automatic number plate recognition	Implementation of Python OpenCV package and other libraries, and use of 20×20 px images	K-Nearest Neighbors (KNN)	The trained model demonstrated an overall classification accuracy of 95% in recognizing number plates of varying sizes, orientations, and shapes across different regions worldwide.	[122]

**Table 16:** Vehicles-related data used in V2X.

Field	Data	Purpose	Refs.
V2V V2I	X-, Y-, and Z-axis acceleration data along with latitude and longitude	To adjust vehicle parameters according to surrounding vehicles and their speeds and eliminate their influence	[27,36,52]
V2X	Position and orientation described by six degrees of freedom and other sensor data, including accelerometers, gyroscopes, magnetometers, camera systems, radars, and Global Navigation Satellite Systems (GNSSs)	To improve the mobility experience of C-ITS in terms of efficiency, safety, and comfort, minimization of human-controlled driving	[1]
V2V V2I	Video, frames, and photos	Detection and prediction using ML models of vehicles, road signs, and obstacles	[37]
V2V V2N	(Relative) Speed, RPM, heading, current action, brake status, and GPS coordinates of vehicle location	Data are used for auxiliary means to achieve better dynamic adjustment between vehicles and to determine any crash risk of the subject vehicle	[2,32,92,123]
V2V V2I V2N	The metadata, preamble (for synchronization) data, network ID, node ID, cyclic redundancy check (CRC), and time stamps	To ensure that all systems correctly understand the start of data transmission, the identification readings are required	[27,92]
V2V V2I V2N	Diagnostic parameters in assessing the technical state of automatic vehicles: work process parameters; parameters of associated processes (vibration, noise); geometrics parameters (clearances, freewheeling, misalignment); and other vehicle telematics data like maintenance requirements and servicing	To enhance traffic safety by warning the driver about mere defects or not allowing automatic driving if there are severe defects	[12,119,141]

**Table 17:** Infrastructure-related data used in V2X.

Field	Data	Purpose	Refs.
V2I	Video, frames, photos, and statistical-numerical information	Detection and prediction using ML models of empty or best parking place	[8]
V2X	Road service data: accident and collision warnings, traffic information, information on traffic jams, or warning of an approaching rescue vehicle	Represent and inform other drivers, including vulnerable road users (pedestrians and cyclists), about warnings, e.g., emergency brake lights, and rerouting suggestions by combining cooperative sensing	[81,126]
V2I V2D	Identification code unique to each sign	The traveling vehicle broadcasts requests for the tags’ identifications by RFID and an identification code unique to each sign is transmitted back	[31]
V2I V2D	Identification code unique to each road part	The traveling vehicle broadcasts requests for the tags’ identifications by RFID and an identification code unique to each road part, e.g., in tunnels, is transmitted back	[142]
V2I	Information on electronic license plates, such as Electronic Product Code (EPC) and phase difference of the backscatter signals	To detect different traffic violations of corresponding vehicle drivers	[68]
V2I	Numerical information about available or occupied parking places	Keep-alive message transmissions, where the parking status is periodically sent if the parking lot status does not change	[49,65]
V2I	Sensor mesh information	A mesh network where each sensor is connected to every other sensor and the information is transmitted to the base station	[30,61]
V2I	Image information of vehicle’s plate.	An image of the vehicle’s plate information is converted into text, then it is sent	[143]
V2I	Ultrasonic sensors, light sensors, magnetic sensors, or accelerometer readings	To detect user and vehicle statuses in a parking place	[15,62]
V2I	RFID ID and timestamp	RFID tag detects and sends arrival time and information about the car or driver (license card or car plate)	[16]

**Table 18:** Other related data used in V2X.

Field	Data	Purpose	Refs.
V2G	Load profile of each (sub-)station, information about each EV, such as the availability and state of charge level of battery, actual power grid loading, target loading, and number of grid connections	To optimize the performance of multiple charging EVs connected to the grid	[117]
V2P	Image of pedestrian and corresponding data	Images are taken from a vehicle, converted into coordinates, and sent to the other vehicle together with the first vehicle’s position	[33]
V2X	Pedestrian position and information concerning its movement, latitudes and longitudes of the smartphone and vehicle, and the moving vectors of the smartphone and the vehicle	For vehicle and pedestrian safety applications to control the vehicle, several essential vehicle parameters, avoidance of severe or deadly collisions, and rerouting vehicles	[19]
V2P V2D	RFID tag’s unique ID, timestamp, and geographical coordinates of the parked car	Uses data regarding when the tag was read and when and where the person left the car for medical purposes or to detect a wandering person	[67]
V2X	The secret key of binary sequence/shared key encryption	For security to avoid information leakage and interruption during transmission using a lower power signal	[144]
V2X	Transition time, device ID, device ID of transmitter of last received message, and reception time of last received message (in-network time)	Uses this safety-related data in the form of low-power beacons to protect data transfer against stationary roadside attackers	[7]
V2X	Common Awareness Messages (CAM) or Basic Safety Messages (BSMs)	These are defined as a broadcast message to avoid packet loss and contain vital, safety-related information: location, speed, heading, and general operation details	[32]
V2V V2I V2N	Weather attributes: temperature sensor data, humidity, moisture, precipitation, visibility, wind, etc. of specific coordinates from the cloud; road characteristics: speed limit, soft or hard turns, highway exits, bridges, etc.; intermediate waypoints where the car needs to reduce its speed: road friction measurements and surface temperature	Based on real-time data, systems inform the driver about a recommended speed that the vehicle should adapt to or automatically reduce to in extreme situations in order to avoid dangerous driving and accidents. To enhance road traffic safety by exchanging real-time or updated weather and traffic data using VANET protocols	[12,14]

## Data Availability

No new data were created or analyzed in this study. Data sharing is not applicable to this article.

## References

[1] Brambilla M., Combi L., Matera A., Tagliaferri D., Nicoli M., Spagnolini U. (2020). Sensor-Aided V2X Beam Tracking for Connected Automated Driving: Distributed Architecture and Processing Algorithms. Sensors.

[2] Kwon D., Park S., Ryu J.-T. (2017). A Study on Big Data Thinking of the Internet of Things-Based Smart-Connected Car in Conjunction with Controller Area Network Bus and 4G-Long Term Evolution. Symmetry.

[3] Ahangar M.N., Ahmed Q.Z., Khan F.A., Hafeez M. (2021). A Survey of Autonomous Vehicles: Enabling Communication Technologies and Challenges. Sensors.

[4] Wang J., Shao Y., Ge Y., Yu R. (2019). A Survey of Vehicle to Everything (V2X) Testing. Sensors.

[5] Arena F., Pau G., Severino A. (2020). An Overview on the Current Status and Future Perspectives of Smart Cars. Infrastructures.

[6] Liu C., Zhou C., Cao W., Li F., Jia P. (2020). A Novel Design and Implementation of Autonomous Robotic Car Based on ROS in Indoor Scenario. Robotics.

[7] Kim H., Kim T. (2019). Vehicle-to-Vehicle (V2V) Message Content Plausibility Check for Platoons through Low-Power Beaconing. Sensors.

[8] Ameen H.A., Mahamad A.K., Saon S., Nor D.M., Ghazi K. (2019). A Review on Vehicle to Vehicle Communication System Applications. Indones. J. Electr. Eng. Comput. Sci..

[9] Luu D.L., Lupu C., Chirita D. Design and Development of Smart Cars Model for Autonomous Vehicles in a Platooning. Proceedings of the International Conference on Engineering of Modern Electric Systems (EMES).

[10] Sobanjo J.O. (2019). Civil Infrastructure Management Models for the Connected and Automated Vehicles Technology. Infrastructures.

[11] Barriga J.J., Sulca J., Luis J.L., Ulloa A., Portero D., Andrade R., Guun S.Y. (2019). Smart Parking: A Literature Review from the Technological Perspective. Appl. Sci..

[12] Tahir M.N., Leviäkangas P., Katz M. (2022). Connected Vehicles: V2V and V2I Road Weather and Traffic Communication Using Cellular Technologies. Sensors.

[13] Biyik C., Allam Z., Pieri G., Moroni D., O’fraifer M., O’connell E., Olariu S., Khalid M. (2021). Smart Parking Systems: Reviewing the Literature, Architecture and Ways Forward. Smart Cities.

[14] Galanis I., Anagnostopoulos I., Gurunathan P., Burkard D. (2019). Environmental-Based Speed Recommendation for Future Smart Cars. Futur. Internet.

[15] Park S. (2021). D-Park: User-Centric Smart Parking System over Ble-Beacon Based Internet of Things. Electronics.

[16] Tsiropoulou E.E., Baras J.S., Papavassiliou S., Sinha S. (2017). RFID-Based Smart Parking Management System. Cyber-Phys. Syst..

[17] Harighi T., Bayindir R., Padmanaban S., Mihet-Popa L., Hossain E. (2018). An Overview of Energy Scenarios, Storage Systems and the Infrastructure for Vehicle-to-Grid Technology. Energies.

[18] Vadi S., Bayindir R., Colak A.M., Hossain E. (2019). A Review on Communication Standards and Charging Topologies of V2G and V2H Operation Strategies. Energies.

[19] Arena F., Pau G., Severino A. (2020). V2X Communications Applied to Safety of Pedestrians and Vehicles. J. Sens. Actuator Netw..

[20] Jing P., Huang W., Chen L. (2017). Car-to-Pedestrian Communication Safety System Based on the Vehicular Ad-Hoc Network Environment: A Systematic Review. Information.

[21] Goikoetxea-Gonzalez J., Casado-Mansilla D., López-De-ipiña D. (2022). The Role of IoT Devices in Sustainable Car Expenses in the Context of the Intelligent Mobility: A Comparative Approach. Appl. Sci..

[22] Storck C.R., Duarte-Figueiredo F. (2019). A 5G V2X Ecosystem Providing Internet of Vehicles. Sensors.

[23] Kumar V., Ahmad M., Mishra D., Kumari S., Khan M.K. (2020). RSEAP: RFID Based Secure and Efficient Authentication Protocol for Vehicular Cloud Computing. Veh. Commun..

[24] Chen C.H., Lee C.R., Lu W.C.H. (2017). Smart In-Car Camera System Using Mobile Cloud Computing Framework for Deep Learning. Veh. Commun..

[25] Tong W., Hussain A., Bo W.X., Maharjan S. (2019). Artificial Intelligence for Vehicle-To-Everything: A Survey. IEEE Access.

[26] Kiela K., Barzdenas V., Jurgo M., Macaitis V., Navickas R. (2020). Review of V2X–IoT Standards and Frameworks for ITS Applications. Appl. Sci..

[27] Haque K.F., Abdelgawad A., Yanambaka V.P., Yelamarthi K. (2020). Lora Architecture for V2x Communication: An Experimental Evaluation with Vehicles on the Move. Sensors.

[28] Almeida J., Rufino J., Alam M., Ferreira J. (2019). A Survey on Fault Tolerance Techniques for Wireless Vehicular Networks. Electronics.

[29] Mihalj T., Li H., Babić D., Lex C., Jeudy M., Zovak G., Babić D., Eichberger A. (2022). Road Infrastructure Challenges Faced by Automated Driving: A Review. Appl. Sci..

[30] Bachani M., Qureshi U.M., Shaikh F.K. (2016). Performance Analysis of Proximity and Light Sensors for Smart Parking. Procedia Comput. Sci..

[31] García Oya J.R., Martín Clemente R., Hidalgo Fort E., González Carvajal R., Muñoz Chavero F. (2018). Passive RFID-Based Inventory of Traffic Signs on Roads and Urban Environments. Sensors.

[32] Wang J., Zheng Q., Mei F., Deng W., Ge Y. (2019). A Novel Method to Enable the Awareness Ability of Non-V2v-Equipped Vehicles in Vehicular Networks. Sensors.

[33] Haselhoff A., Hoehmann L., Kummert A., Nunn C., Meuter M., Mueller-Schneiders S. Multi-Camera Pedestrian Detection by Means of Track-to-Track Fusion and Car2Car Communication. Proceedings of the International Conference on Computer Vision Theory and Applications.

[34] Shen W.H., Tsai H.M. Testing Vehicle-to-Vehicle Visible Light Communications in Real-World Driving Scenarios. Proceedings of the 2017 IEEE Vehicular Networking Conference (VNC).

[35] Wang J.G., Zhou L., Pan Y., Lee S., Song Z., Han B.S., Saputra V.B. Appearance-Based Brake-Lights Recognition Using Deep Learning and Vehicle Detection. Proceedings of the 2016 IEEE Intelligent Vehicles Symposium (IV).

[36] Lyu F., Zhu H., Cheng N., Zhou H., Xu W., Li M., Shen X. (2020). Characterizing Urban Vehicle-to-Vehicle Communications for Reliable Safety Applications. IEEE Trans. Intell. Transp. Syst..

[37] Masiero A., Toth C., Gabela J., Retscher G., Kealy A., Perakis H., Gikas V., Grejner-Brzezinska D. (2021). Experimental Assessment of UWB and Vision-Based Car Cooperative Positioning System. Remote Sens..

[38] Ammour N., Alhichri H., Bazi Y., Benjdira B., Alajlan N., Zuair M. (2017). Deep Learning Approach for Car Detection in UAV Imagery. Remote Sens..

[39] Plattner M., Ostermayer G. (2021). Undersampled Differential Phase Shift On–Off Keying for Visible Light Vehicle-to-Vehicle Communication. Appl. Sci..

[40] Iacobescu C., Oltean G., Florea C., Burtea B. (2022). Unified Interplanetary Smart Parking Network for Maximum End-User Flexibility. Sensors.

[41] Farley A., Ham H. (2021). Hendra Real Time IP Camera Parking Occupancy Detection Using Deep Learning. Procedia Comput. Sci..

[42] Shariff W., Dilmaghani M.S., Kielty P., Moustafa M., Lemley J., Corcoran P. (2024). Event Cameras in Automotive Sensing: A Review. IEEE Access.

[43] von Arnim A., Lecomte J., Borras N.E., Woźniak S., Pantazi A. (2024). Dynamic Event-Based Optical Identification and Communication. Front. Neurorobot..

[44] Carmichael S., Buchan A., Ramanagopal M., Ravi R., Jan R.O. (2024). Dataset and Benchmark: Novel Sensors for Autonomous Vehicle Perception. arXiv.

[45] Jakab D., Deegan B.M., Sharma S., Grua E.M., Horgan J., Ward E., van de Ven P., Scanlan A., Eising C. (2024). Surround-View Fisheye Optics in Computer Vision and Simulation: Survey and Challenges. IEEE Trans. Intell. Transp. Syst..

[46] Yogamani S., Unger D., Narayanan V., Kumar V.R. (2024). DaF-BEVSeg: Distortion-Aware Fisheye Camera Based Bird’s Eye View Segmentation with Occlusion Reasoning. https://arxiv-sanity-lite.com/?rank=pid&pid=2404.06352.

[47] Koplon L.W., Nessaee A.G., Choi A., Mentoza A., Villasana M. (2024). Cyclops: Binding a Vehicle ’s Digital Identity to Its Physical Trajectory Using Monocular Cameras. Symp. Veh. Secur. Priv..

[48] Li Y., Moreau J., Ibanez-Guzman J. (2022). Emergent Visual Sensors for Autonomous Vehicles. IEEE Trans. Intell. Transp. Syst..

[49] Rodić L.D., Perković T., Županović T., Šolić P. (2020). Sensing Occupancy through Software: Smart Parking Proof of Concept. Electronics.

[50] Lou L., Zhang J., Xiong Y., Jin Y. (2019). An Improved Roadside Parking Space Occupancy. Sensors.

[51] Perković T., Šolić P., Zargariasl H., Čoko D., Rodrigues J.J.P.C. (2020). Smart Parking Sensors: State of the Art and Performance Evaluation. J. Clean. Prod..

[52] Xu R., Liu H., Wang H.H. Design of Smart Car Networking System Based on Multi-Sensor Network. Proceedings of the 2021 6th International Conference on Inventive Computation Technologies (ICICT).

[53] Hossen M.I., Michael G.K.O., Connie T., Lau S.H., Hossain F. (2019). Smartphone-Based Context Flow Recognition for Outdoor Parking System with Machine Learning Approaches. Electronics.

[54] Doniec R.J., Sieciński S., Duraj K.M., Piaseczna N.J., Mocny-Pachońska K., Tkacz E.J. (2020). Recognition of Drivers’ Activity Based on 1D Convolutional Neural Network. Electronics.

[55] Celaya-Padilla J.M., Galván-Tejada C.E., López-Monteagudo F.E., Alonso-González O., Moreno-Báez A., Martínez-Torteya A., Galván-Tejada J.I., Arceo-Olague J.G., Luna-García H., Gamboa-Rosales H. (2018). Speed Bump Detection Using Accelerometric Features: A Genetic Algorithm Approach. Sensors.

[56] Zhang S., Wang C., Lin L., Wen C., Yang C., Zhang Z., Li J. (2019). Automated Visual Recognizability Evaluation of Traffic Sign Based on 3D LiDAR Point Clouds. Remote Sens..

[57] Javanmardi M., Song Z., Qi X. (2021). A Fusion Approach to Detect Traffic Signs Using Registered Color Images and Noisy Airborne LiDAR Data. Appl. Sci..

[58] Schinkel W., van der Sande T., Nijmeijer H. (2021). State Estimation for Cooperative Lateral Vehicle Following Using Vehicle-to-Vehicle Communication. Electronics.

[59] Li J.T., Li Z.E., Li X.F., Yang Y.J., Xiao J. (2020). A Simple and Efficient Algorithm Design for Improving the Infrared Tracking Accuracy of Smart Cars. Procedia Comput. Sci..

[60] Sviatov K., Yarushkina N., Kanin D., Rubtcov I., Jitkov R., Mikhailov V., Kanin P. (2021). Functional Model of a Self-Driving Car Control System. Technologies.

[61] Bagula A., Castelli L., Zennaro M. (2015). On the Design of Smart Parking Networks in the Smart Cities: An Optimal Sensor Placement Model. Sensors.

[62] Alshehri F., Almawgani A.H.M., Alqahtani A., Alqahtani A. Smart Parking System for Monitoring Cars and Wrong Parking. Proceedings of the 2019 2nd International Conference on Computer Applications & Information Security (ICCAIS).

[63] Panganiban E.B., Dela Cruz J.C. RFID-Based Vehicle Monitoring System. Proceedings of the 2017 IEEE 9th International Conference on Humanoid, Nanotechnology, Information Technology, Communication and Control, Environment, and Management (HNICEM).

[64] Roy D., Li Y., Jian T., Tian P., Chowdhury K., Ioannidis S. (2023). Multi-Modality Sensing and Data Fusion for Multi-Vehicle Detection. IEEE Trans. Multimed..

[65] Hilmani A., Maizate A., Hassouni L. (2018). Designing and Managing a Smart Parking System Using Wireless Sensor Networks. J. Sens. Actuator Netw..

[66] Salah B. (2020). Design, Simulation, and Performance-Evaluation-Based Validation of a Novel RFID-Based Automatic Parking System. Simulation.

[67] Griggs W.M., Verago R., Naoum-Sawaya J., Ordonez-Hurtado R.H., Gilmore R., Shorten R.N. (2018). Localizing Missing Entities Using Parked Vehicles: An RFID-Based System. IEEE Internet Things J..

[68] Zhang Y., Ma Y., Liu K., Wang J., Li S. RFID Based Vehicular Localization for Intelligent Transportation Systems. Proceedings of the 2019 IEEE International Conference on RFID Technology and Applications (RFID-TA).

[69] Barriga J.J., Sulca J., León J., Ulloa A., Portero D., García J., Yoo S.G. (2020). A Smart Parking Solution Architecture Based on LoRaWAN and Kubernetes. Appl. Sci..

[70] Coulibaly M., Errami A., Belkhala S., Medromi H. (2021). A Live Smart Parking Demonstrator: Architecture, Data Flows, and Deployment. Energies.

[71] Celaya-Echarri M., Azpilicueta L., Lopez-Iturri P., Picallo I., Aguirre E., Astrain J.J., Villadangos J., Falcone F. (2020). Radio Wave Propagation and WSN Deployment in Complex Utility Tunnel Environments. Sensors.

[72] Fahim A., Hasan M., Chowdhury M.A. (2021). Smart Parking Systems: Comprehensive Review Based on Various Aspects. Heliyon.

[73] Granda F., Azpilicueta L., Vargas-Rosales C., Lopez-Iturri P., Aguirre E., Astrain J.J., Villandangos J., Falcone F. (2018). Deterministic Propagation Modeling for Intelligent Vehicle Communication in Smart Cities. Proceedings.

[74] Granda F., Azpilicueta L., Vargas-Rosales C., Lopez-Iturri P., Aguirre E., Astrain J.J., Villandangos J., Falcone F. (2017). Characterization of Radio Propagation Channel in Urban Vehicle to Infrastructure Environments to Support WSNs. Proceedings.

[75] Correa A., Boquet G., Morell A., Vicario J.L. (2017). Autonomous Car Parking System through a Cooperative Vehicular Positioning Network. Sensors.

[76] Ledergerber A., D’Andrea R. (2020). A Multi-Static Radar Network with Ultra-Wideband Radio-Equipped Devices. Sensors.

[77] Swatha M., Pooja K. Smart Car Parking with Monitoring System. Proceedings of the IEEE International Conference on System, Computation, Automation and Networking (ICSCAN).

[78] Baral P. (2024). Report on Preparation of Topographical Map of Dhulikhel Kavre Nepal Using Unmanned Air Vehicle (UAV).

[79] Li X., Huang J., Li X., Shen Z., Han J., Li L., Wang B. (2022). Review of PPP–RTK: Achievements, Challenges, and Opportunities. Satell. Navig..

[80] Li X., Xu Q., Li X., Xin H., Yuan Y., Shen Z., Zhou Y. (2024). Improving PPP-RTK-Based Vehicle Navigation in Urban Environments via Multilayer Perceptron-Based NLOS Signal Detection. GPS Solut..

[81] Spahiu C.S., Stanescu L., Brezovan M., Petcusin F. Lifi Technology Feasibility Study for Car-2-Car Communication. Proceedings of the 2020 21th International Carpathian Control Conference (ICCC).

[82] Sharma T., Debaque B., Duclos N., Chehri A., Kinder B., Fortier P. (2022). Deep Learning-Based Object Detection and Scene Perception under Bad Weather Conditions. Electronics.

[83] Shipman R., Waldron J., Naylor S., Pinchin J., Rodrigues L., Gillott M. (2020). Where Will You Park? Predicting Vehicle Locations for Vehicle-to-grid. Energies.

[84] Balfaqih M., Jabbar W., Khayyat M., Hassan R. (2021). Design and Development of Smart Parking System Based on Fog Computing and Internet of Things. Electronics.

[85] Bertipaglia A., Alirezaei M., Happee R., Shyrokau B. (2023). An Unscented Kalman Filter-Informed Neural Network for Vehicle Sideslip Angle Estimation. Res. Output Contrib. J..

[86] Du X., Hu X.B., Hu J.S., Sun Z.D. (2023). An Adaptive Interactive Multi-Model Navigation Method Based on UUV. Ocean Eng..

[87] Chen B., Dang L., Zheng N., Principe J.C. (2023). Additional Topics in Kalman Filtering Under Information Theoretic Criteria. Kalman Filter. Under Inf. Theor. Criteria.

[88] Lin M., Yoon J., Kim B. (2020). Self-Driving Car Location Estimation Based on a Particle-Aided Unscented Kalman Filter. Sensors.

[89] Tao B., Wu H., Gong Z., Yin Z., Ding H. (2021). An RFID-Based Mobile Robot Localization Method Combining Phase Difference and Readability. IEEE Trans. Autom. Sci. Eng..

[90] Zhang F., Hinz G., Clarke D., Knoll A. Vehicle-Infrastructure Localization Based on the SME Filter. Proceedings of the 2015 IEEE 18th International Conference on Intelligent Transportation Systems-(ITSC 2015).

[91] Bai L., Zhao Y., Huang X. A Near Sensor Edge Computing System for Point Cloud Semantic Segmentation. Proceedings of the 2022 IEEE International Symposium on Circuits and Systems (ISCAS).

[92] Chandra K., Rayamajhi A., Chowdhury M., Bhavsar P., Martin J. (2016). Vehicle-to-Vehicle (V2V) and Vehicle-to-Infrastructure (V2I) Communication in a Heterogeneous Wireless Network—Performance Evaluation. Transp. Res. Part C.

[93] Lyu N., Wen J., Wu C. (2021). Novel Time-Delay Side-Collision Warning Model at Non-Signalized Intersections Based on Vehicle-to-Infrastructure Communication. Int. J. Environ. Res. Public Health.

[94] Mir Z.H., Filali F. (2022). C-ITS Applications, Use Cases and Requirements for V2X Communication Systems—Threading through IEEE 802.11p to 5G. Towards a Wireless Connected World: Achievements and New Technologies.

[95] Li T., Wu X., Zhang J. (2020). Time Series Clustering Model Based on DTW for Classifying Car Parks. Algorithms.

[96] Ouali Y., Hudelot C., Tami M. (2020). An Overview of Deep Semi-Supervised Learning. arXiv.

[97] Kiran B.R., Sobh I., Talpaert V., Mannion P., Sallab A.A.A., Yogamani S., Perez P. (2022). Deep Reinforcement Learning for Autonomous Driving: A Survey. IEEE Trans. Intell. Transp. Syst..

[98] Ni J., Chen Y., Chen Y., Zhu J., Ali D., Cao W. (2020). A Survey on Theories and Applications for Self-Driving Cars Based on Deep Learning Methods. Appl. Sci..

[99] Xu Q., Wang B., Zhang F., Regani D.S., Wang F., Ray Liu K.J. (2020). Wireless AI in Smart Car: How Smart a Car Can Be?. IEEE Access.

[100] Khan M.Z., Sarkar A., Ghandorh H., Driss M., Boulila W. (2022). Information Fusion in Autonomous Vehicle Using Artificial Neural Group Key Synchronization. Sensors.

[101] Manase D.K., Zainuddin Z., Syarif S., Jaya A.K. Car Detection in Roadside Parking for Smart Parking System Based on Image Processing. Proceedings of the 2020 International Seminar on Intelligent Technology and Its Applications (ISITIA).

[102] Moaga M., Chunling T., Owolawi P. (2024). Vision-Based Multi-Detection and Tracking of Vehicles Using the Convolutional Neural Network Model YOLO. Lect. Notes Netw. Syst..

[103] Syed A.M., Devisurya V., Gavin S., Kamal A. (2024). Enhanced Number Plate Recognition for Restricted Area Access Control Using Deep Learning Models and EasyOCR Integration. SSRN Electron. J..

[104] Singh R., Goyal S., Agarwal S., Upadhyay S. (2024). Evaluating the Performance of Ensembled YOLOv8 Variants in Smart Parking Applications for Vehicle Detection and License Plate Recognition under Varying Lighting Conditions. Preprints.

[105] Cao J., Song C., Song S., Peng S., Wang D., Shao Y., Xiao F. (2020). Front Vehicle Detection Algorithm for Smart Car Based on Improved SSD Model. Sensors.

[106] Cao J., Song C., Peng S., Xiao F., Song S. (2019). Improved Traffic Sign Detection and Recognition Algorithm for Intelligent Vehicles. Sensors.

[107] Chou S.Y., Dewabharata A., Zulvia F.E. (2022). Dynamic Space Allocation Based on Internal Demand for Optimizing Release of Shared Parking. Sensors.

[108] Liu Q., Li Z., Yuan S., Zhu Y., Li X. (2021). Review on Vehicle Detection Technology for Unmanned Ground Vehicles. Sensors.

[109] Gao G., Wüthrich M.V. (2019). Convolutional Neural Network Classification of Telematics Car Driving Data. Risks.

[110] Dodia A., Kumar S. A Comparison of YOLO Based Vehicle Detection Algorithms. Proceedings of the Artificial Intelligence and Applications (ICAIA), International Conference on, Technology Conference (ATCON-1).

[111] Fan P., Guo J., Zhao H., Wijnands J.S., Wang Y. (2019). Car-Following Modeling Incorporating Driving Memory Based on Autoencoder and Long Short-Term Memory Neural Networks. Sustainability.

[112] Yang F., Yang D., He Z., Fu Y., Jiang K. (2020). Automobile Fine-Grained Detection Algorithm Based on Multi-Improved YOLOv3 in Smart Streetlights. Algorithms.

[113] Vikruthi S., Archana M., Tanguturi R.C. (2023). A Novel Framework for Vehicle Detection and Classification Using Enhanced YOLO-v7 and GBM to Prioritize Emergency Vehicle. Int. J. Intell. Syst. Appl. Eng..

[114] Tran Ngoc H., Da Quach L., Hoang Nguyen K., Hoang Nguyen K., Khanh Hua H., Vu Nhu Nguyen H., Quach L.-D. (2023). Optimizing YOLO Performance for Traffic Light Detection and End-to-End Steering Control for Autonomous Vehicles in Gazebo-ROS2. Artic. Int. J. Adv. Comput. Sci. Appl..

[115] Chang C.C., Tsai J., Lin J.H., Ooi Y.M. (2021). Autonomous Driving Control Using the DDPG and RDPG Algorithms. Appl. Sci..

[116] Yang Q., Lu T., Zhou H. (2022). A Spatio-Temporal Motion Network for Action Recognition Based on Spatial Attention. Entropy.

[117] Tan K.M., Ramachandaramurthy V.K., Yong J.Y., Padmanaban S., Mihet-Popa L., Blaabjerg F. (2017). Minimization of Load Variance in Power Grids-Investigation on Optimal Vehicle-to-Grid Scheduling. Energies.

[118] Hadiwardoyo S.A., Calafate C.T., Cano J.C., Krinkin K., Klionskiy D., Hernández-Orallo E., Manzoni P. (2020). Three Dimensional UAV Positioning for Dynamic UAV-to-Car Communications. Sensors.

[119] Kowalik B., Szpyrka M. (2019). An Entropy-Based Car Failure Detection Method Based on Data Acquisition Pipeline. Entropy.

[120] Qiong W., Shuai S., Ziyang W., Qiang F., Pingyi F., Cui Z. (2023). Towards V2I Age-Aware Fairness Access: A DQN Based Intelligent Vehicular Node Training and Test Method. Chin. J. Electron..

[121] Awan F.M., Saleem Y., Minerva R., Crespi N. (2020). A Comparative Analysis of Machine/Deep Learning Models for Parking Space Availability Prediction. Sensors.

[122] Ahmed A.A., Ahmed S. (2021). A Real-Time Car Towing Management System Using Ml-Powered Automatic Number Plate Recognition. Algorithms.

[123] Wei Y., Chen J., Hwang S.H. (2018). Adjacent Vehicle Number-Triggered Adaptive Transmission for V2V Communications. Sensors.

[124] Haider S., Abbas Z.H., Abbas G., Waqas M., Tu S., Zhao W. (2020). A Novel Cross-Layer V2V Architecture for Direction-Aware Cooperative Collision Avoidance. Electronics.

[125] Flanagan S.K., Tang Z., He J., Yusoff I. (2021). Investigating and Modeling of Cooperative Vehicle-to-Vehicle Safety Stopping Distance. Futur. Internet.

[126] Kosmanos D., Chaikalis C., Savvas I.K. (2022). 3GPP 5G V2X Scenarios: Performance of QoS Parameters Using Turbo Codes. Telecom.

[127] Hossen M.S., Kabir A.F.M.S., Khan R.H., Azfar A. (2010). Interconnection between 802.15.4 Devices and IPv6: Implications and Existing Approaches. Int. J. Comput. Sci. Issues.

[128] Gagliardi G., Lupia M., Cario G., Tedesco F., Gaccio F.C., Lo Scudo F., Casavola A. (2020). Advanced Adaptive Street Lighting Systems for Smart Cities. Smart Cities.

[129] Etxaniz J., Aranguren G. (2017). Bluetooth Low Power Modes Applied to the Data Transportation Network in Home Automation Systems. Sensors.

[130] Anwar W., Franchi N., Fettweis G. (2019). Physical Layer Evaluation of V2X Communications Technologies. https://www.vodafone-chair.org/pbls/waqar-anwar/On_the_Reliability_of_NR_V2X_and_IEEE_802_11bd.pdf.

[131] Naik G., Member S., Choudhury B. (2019). IEEE 802. 11bd & 5G NR V2X: Evolution of Radio Access Technologies for V2X Communications. IEEE Access.

[132] Bazzi A., Cecchini G., Menarini M., Masini B.M., Zanella A. (2019). Survey and Perspectives of Vehicular Wi-Fi versus Sidelink Cellular-V2X in the 5G Era. Futur. Internet.

[133] Baros J., Martinek R., Jaros R., Danys L., Soustek L. (2019). Development of Application for Control of SMART Parking Lot. IFAC-PapersOnLine.

[134] Tsiknas K., Stamatelos G. (2012). Comparative Performance Evaluation of TCP Variants in WiMAX (and WLANs) Network Configurations. J. Comput. Netw. Commun..

[135] Avcil M.N., Soyturk M., Kantarci B. (2024). Fair and Efficient Resource Allocation via Vehicle-Edge Cooperation in 5G-V2X Networks. Veh. Commun..

[136] Noor-A-Rahim M., Liu Z., Lee H., Khyam M.O., He J., Pesch D., Moessner K., Saad W., Poor H.V. (2022). 6G for Vehicle-to-Everything (V2X) Communications: Enabling Technologies, Challenges, and Opportunities. Proc. IEEE.

[137] Praveen M., Harini V. NB-IOT Based Smart Car Parking System. Proceedings of the 2019 International Conference on Smart Structures and Systems (ICSSS).

[138] Fernández-Caramés T.M., Fraga-Lamas P., Suárez-Albela M., Castedo L. (2017). Reverse Engineering and Security Evaluation of Commercial Tags for RFID-Based IoT Applications. Sensors.

[139] Majumder S., Mandava D.C., Kim J., Javaid A.Y. Multimedia Transmission for V2X Communication over Legacy LTE-A Network Infrastructure-A Performance Evaluation. Proceedings of the 2020 11th IEEE Annual Ubiquitous Computing, Electronics & Mobile Communication Conference (UEMCON).

[140] Scholtes M., Westhofen L., Turner L.R., Lotto K., Schuldes M., Weber H., Wagener N., Neurohr C., Bollmann M.H., Kortke F. (2021). 6-Layer Model for a Structured Description and Categorization of Urban Traffic and Environment. IEEE Access.

[141] Makarova I., Mukhametdinov E., Tsybunov E. (2018). Management of the Reliability of Intelligent Vehicles as a Method to Improve Traffic Safety. Transp. Res. Procedia.

[142] Chen R., Huang X., Zhou Y., Hui Y., Cheng N. (2022). UHF-RFID-Based Real-Time Vehicle Localization in GPS-Less Environments. IEEE Trans. Intell. Transp. Syst..

[143] Luque-Vega L.F., Michel-Torres D.A., Lopez-Neri E., Carlos-Mancilla M.A., González-Jiménez L.E. (2020). Iot Smart Parking System Based on the Visual-Aided Smart Vehicle Presence Sensor: SPIN-V. Sensors.

[144] Han B., Peng S., Wu C., Wang X., Wang B. (2020). Lora-Based Physical Layer Key Generation for Secure V2V/V2I Communications. Sensors.

